# Natural variation of gene models in *Drosophila melanogaster*

**DOI:** 10.1186/s12864-015-1415-6

**Published:** 2015-03-17

**Authors:** Yerbol Z Kurmangaliyev, Alexander V Favorov, Noha M Osman, Kjong-Van Lehmann, Daniel Campo, Matthew P Salomon, John Tower, Mikhail S Gelfand, Sergey V Nuzhdin

**Affiliations:** University of Southern California, Los Angeles, CA USA; Institute for Information Transmission Problems (Kharkevich Institute), Moscow, Russia; Johns Hopkins University School of Medicine, Baltimore, MD USA; Vavilov Institute of General Genetics, Moscow, Russia; Research Institute of Genetics and Selection of Industrial Microorganisms, Moscow, Russia; National Research Center, Dokki, Giza Egypt; Memorial Sloan Kettering Cancer Center, Zuckerman Research Center, New York, NY USA; Lomonosov Moscow State University, Moscow, Russia; Saint Petersburg Polytechnical University, St Petersburg, Russia

**Keywords:** Drosophila, Splicing, Natural variation, Quantitative trait loci

## Abstract

**Background:**

Variation within splicing regulatory sequences often leads to differences in gene models among individuals within a species. Two alleles of the same gene may express transcripts with different exon/intron structures and consequently produce functionally different proteins. Matching genomic and transcriptomic data allows us to identify putative regulatory variants associated with changes in splicing patterns.

**Results:**

Here we analyzed natural variation of splicing patterns in the transcriptomes of 81 natural strains of *Drosophila melanogaster* with known genotypes. We identified dozens of genotype-specific splicing patterns associated with putative *cis*-splicing quantitative trait loci (sQTL). The majority of changes can be explained by mutations in splice sites. Allelic-imbalance in splicing patterns confirmed that the majority are regulated mainly by *cis*-genetic effects. Remarkably, allele-specific splicing changes often lead to qualitative changes in gene models, yielding many isoforms not previously annotated. The observed alterations are typically outside protein-coding regions or affect only very short protein segments.

**Conclusions:**

Overall, the sets of gene models appear to be flexible within *D. melanogaster* populations. The observed variation in splicing patterns are predicted to have limited effects on the encoded protein sequences. To our knowledge, this is the first sQTL mapping study in *Drosophila*.

**Electronic supplementary material:**

The online version of this article (doi:10.1186/s12864-015-1415-6) contains supplementary material, which is available to authorized users.

## Background

Splicing is a complex process regulated by multiple *trans*-factors in conjunction with *cis*-regulatory signals. The latter determine the precise recognition of exon/intron boundaries and the structure of mature transcripts [1]. Mutations in *cis*-regulatory motifs may lead to changes in splicing patterns and hence strongly influence gene function. Disruptions of splicing patterns are commonly considered as loss-of-function variants and indeed they are frequently associated with genetic diseases [2-4]. Surprisingly, multiple studies have reported widespread natural variation in splicing patterns among healthy individuals. Two alleles of the same gene may lead to the expression of transcripts with different exon/intron structure [5-10]. In turn, the analysis of associations between variation in splicing patterns and whole genome sequences enables the discovery of putative regulatory variants, splicing quantitative trait loci (sQTL) [11-14].

Previously, we have analyzed a large set of splice-site disrupting polymorphisms observed in modern human populations [15]. Although mutations at canonical splicing dinucleotides are supposed to cause dramatic changes in splicing patterns, our study showed that the majority of these mutations had minor functional effects. Most polymorphisms affected rarely used and weakly conserved isoforms. The resulting changes in splicing patterns had little effect on the encoded protein structure [15]. However, these observations were solely based on predicted disruptions of known annotated isoforms. We did not have the capacity to annotate and analyze exact molecular outcomes of changes in gene models at that time.

A straightforward way to detect a new splicing pattern is to observe it in individual transcriptomic data. Here we analyzed allele-specific splicing patterns in matching genomic and transcriptomic data for 81 *D. melanogaster* hybrid strains generated by crosses of inbred strains from natural populations to a single inbred reference strain. We identified dozens of putative regulatory variants associated with genotype-specific changes in splicing patterns. Notably, a large fraction of the observed changes resulted in previously unannotated gene models. The predicted functional effects of the splicing changes were consistent with our previous conclusions [15] supporting the thesis that natural variation in splicing patterns is likely to have only modest direct effect on the encoded protein sequences.

## Results

### Genotype-specific transcriptomes of *D. melanogaster* lines

**Figure 1:**
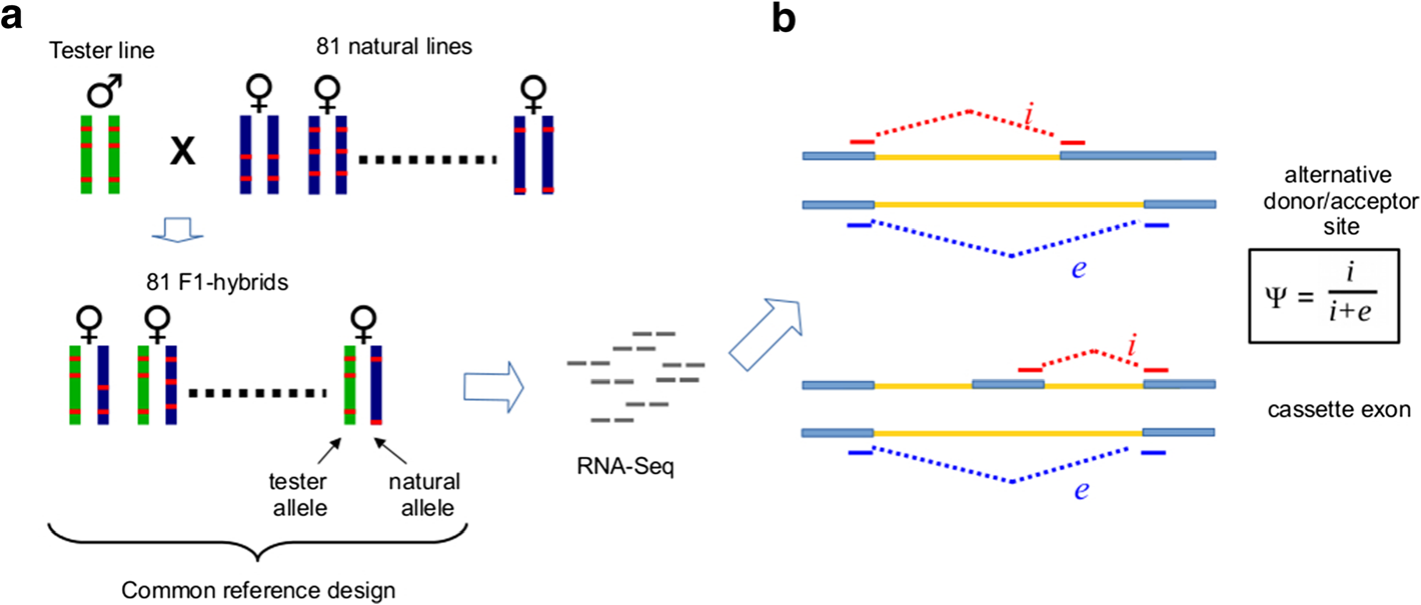
**Genotype-specific transcriptomes. a:** Common reference design. 81 inbred lines, which were derived from natural populations **(natural lines)**, were crossed with one common line **(tester line)**. Each heterozygous F1-hybrid had the same tester allele **(green bars)** and variable natural allele **(dark blue bars)**. Transcriptomes of these F1-hybrids were sequenced and used to annotate and quantify genotype-specific splicing patterns. **b:** Annotation and quantification of alternative splicing events. We focused on two most common types of alternative splicing events, namely, alternative donor/acceptor sites and cassette exons. The counts of inclusion **(** ***i*** **)** and exclusion **(** ***e*** **)** junction reads were used to quantify each alternative splicing event in each F1-hybrid. **Ψ**-value represented fraction of the longer isoform among two alternate transcripts.

We analyzed a panel of RNA-seq datasets from 81 *D. melanogaster* heterozygotes with known genotypes [Lehmann et al., manuscript submitted]. The flies were crossbred following a common reference design [16], such that multiple inbred strains from natural populations (natural lines) were crossed with a single common tester strain (*w[1118]*). The resulting transcriptomes of F1-hybrids were subsequently sequenced (Figure 1a, see Methods). The measurement of allelic imbalance in a splicing patterns allowed us to partition *cis-* and *trans-* genetic effects ([16], see below). In total, the dataset consisted of 800 million uniquely mapped paired-end RNA-Seq reads. The total coverage varied from 1.3 to 41 million reads per genotype (Additional file 1: Table S1).

Genotype-specific changes in splicing patterns may be both quantitative (i.e. changes in ratios of existing isoforms) and qualitative (i.e. expression of new isoforms). To be able to detect new genotype-specific isoforms, we annotated all alternative splicing events observed in the analyzed dataset. To this end we extended the base set of annotated introns with newly detected splice junctions and used them to annotate and quantify alternative splicing events (see Methods). We concentrated on two most common subtypes, namely, alternative donor/acceptor sites (exon extension/truncation events) and cassette exons (exon inclusion/skipping events) (Figure 1b). Particularly, we did not account for retained introns since any observed intron retention event might stem from contamination of underspliced transcripts [17]. Overall, the dataset consisted of 2744 alternative splicing events, including 2126 cases of alternative splice-site usage and 618 cases of cassette exons (Additional file 1: Table S2).

Alternative splicing events were quantified in each genotype by a Ψ-value (PSI, Percent Spliced In) which is a natural representation of the fraction of one of the isoforms among two major alternate transcripts (see Methods, Figure 1b).

### Genotype-specific splicing events associated with *cis*-sQTL

We ran association tests between the quantitative estimates of splicing patterns (Ψ-values) with single-nucleotide polymorphisms (SNPs) to detect putative regulatory variants. A relatively low sample size (81 genotypes) restricted our power to perform genome-wide association tests, therefore we focused only on variants located in close proximity to the tested alternative splicing events (within 500 bp upstream or downstream). This is a minor concern, as it has previously been shown that splicing patterns tend to be determined mainly by *cis*-variants [11,18,19].

Natural inbred lines used in this study were collected from two North American populations of *D. melanogaster* (from Raleigh, North Carolina [20] and from Winters, California [21]), and a significant level of genome-wide differentiation was detected among the lines. In order to take this into account, we used EMMA [22], a well-established method to test for associations while correcting for confounding effects including the population structure (see Methods).

In total, we performed 120,240 association tests between alternative splicing events and *cis*-SNPs. We used the Benjamini-Hochberg FDR 1% value as a statistical significance threshold. We also filtered significant hits by their effect sizes, which were calculated as the difference between median Ψ-values for two alternate alleles of the tested SNP (ΔΨ). We focused on cases with ΔΨ > 0.1. We detected 24 alternative splicing events that were associated with 67 *cis*-splicing quantitative trait loci (*cis*-sQTL) with ΔΨ > 0.1 (Additional file 1: Table S3). We refer to the alternative splicing events that exhibit changes in Ψ-values associated with sQTL as genotype-specific splicing events. In those cases where changes in splicing were associated with multiple *cis*-SNPs, the SNPs most likely formed LD-blocks.

The majority of detected genotype-specific splicing events had at least one *cis*-sQTL overlapping with splice sites (in 15 of 24 cases) suggesting that these mutations were likely the causative regulatory variants. Splice sites were defined as regions around intron/exon boundaries from −3 to +6 for donor sites and from −15 to +3 for acceptor sites. Moreover, in seven of these cases mutations occurred in canonical splicing dinucleotides of splice sites (GT/AG) and thus undoubtedly can be considered as regulatory SNPs (discussed in detail below). In the remaining nine cases, all associated *cis*-QTL were located outside of splice site regions. They might represent either auxiliary regulatory sites or SNPs that are in LD with undetected causative SNPs.

**Figure 2:**
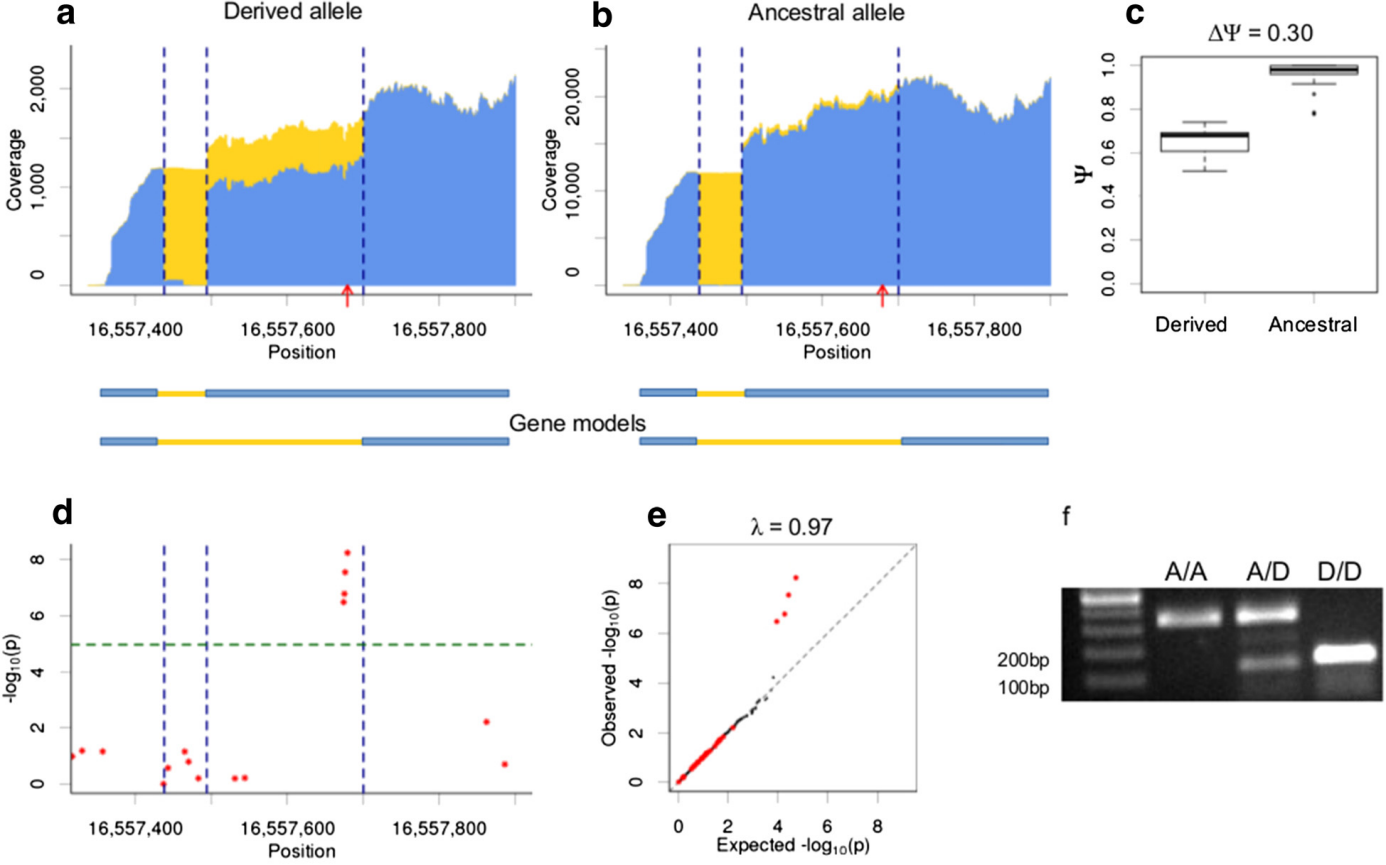
**Genotype-specific alternative acceptor site associated with** ***cis*** **-sQTL (a2016).** A cryptic acceptor site activation and removal of a 206 bp segment that is associated with four *cis*-sQTL. All plots are shown for the most significant *cis*-sQTL. **a-b:** Exon/intron coverage plots of the genotype-specific splicing event are shown for the F1-hybrids that carried derived **(a)** and ancestral **(b)** variants of *cis*-sQTL on natural alleles. Tester alleles of all F1-hybrids carried ancestral variant of *cis*-sQTL. The coverage for each genomic positions is shown separately for exonic **(blue)** and intronic **(yellow)** parts of reads. The coverage from multiple genotypes that carry the same allele is merged. Exon/intron boundaries are marked by **vertical dashed lines**. The position of *cis*-sQTL is shown by the **red arrow**. The alternative gene models are shown under the coverage plots: **blue rectangles** and **yellow lines** correspond to exons and introns, respectively. **c:** The distributions of Ψ-values for alleles of this *cis*-sQTL. **d:** The Manhattan plot of association test log-p-values for all tested *cis*-SNPs. **e:** Q-Q plot for the observed **(y-axis)** and expected **(x-axis)** distributions of association p-values for the tested *cis*-SNPs **(red dots)** and 10000 random SNPs from other chromosomes **(black dots). f:** RT-PCR validation of gene models. The predicted lengths of PCR-products corresponding to the long and short isoforms were 337 bp and 131 bp, respectively. **A/A**: inbred line carrying only ancestral alleles (tester line); **A/D**: F1-hybrid carrying both ancestral and derived alleles; **D/D**: inbred line carrying only derived alleles (natural line).

A representative example of a detected genotype-specific splicing event is shown in Figure 2. We used cumulative exon/intron coverage plots to visualize differences in splicing patterns between two alternative alleles of *cis*-sQTL (Figure 2ab). It should be noticed that we were considering the state of SNP only for the natural alleles. The tester alleles in all F1-hybrids carried the ancestral variant of *cis*-sQTL. In these coverage plots we show the RNA-Seq coverage of each genomic position for both exonic (blue) and intronic (yellow) parts of a read. Coverage from multiple F1-hybrids that carried the same variants of *cis*-sQTL was merged for illustration purposes. The coverage plots were generated for the alleles of the most significant SNP. The distributions of Ψ-values for each F1-hybrid that carried alternate variants of the SNP are shown in Figure 2c.

This example represents activation of a cryptic acceptor site associated with four closely linked SNPs located approximately 20 bp upstream from the activated splice site. This leads to the removal of a 206 bp protein-coding region from the second exon of the gene *CG31205* (S-Peptidase).

To test for potential inflation of false-positive results we performed association tests between detected genotype-specific splicing pattern with 10,000 random SNPs from other chromosomes and calculated genome-wide inflation factor (λ, see Methods). A diagnostic Q-Q plot for the observed and expected distributions of log-p-values for the presented example is shown in Figure 2e. No genome-wide inflation was observed in any of the detected genotypes-specific splicing events (Additional file 1: Table S3).

The expression of the predicted isoforms was validated by RT-PCR (Figure 2f). Validation was performed for one of the F1-hybrids, which carried the derived allele of the most significant *cis*-sQTL, and for the respective inbred parental lines. We could show that the inbred tester line, which carried only ancestral alleles (A/A), expressed the long isoform (337 bp band). The inbred natural line, which carried only derived alleles (D/D), expressed only the short isoform corresponding to the activation of a cryptic splice site (131 bp band). F1-hybrids, which carried both alleles (A/D), expressed both isoforms. The absence of the long isoform in the homozygous D/D-inbred lines implies that in this case we observe a complete switch of isoforms between alternate alleles.

### Genotype-specific splicing events associated with SNPs in canonical splicing dinucleotides

Canonical splicing dinucleotides are the most conserved positions in splice sites (GT/AG). Mutations that occurred at these positions resulted in complete absence of splicing (not-GT/AG alleles). We identified seven *cis*-sQTL in our association tests (see above) that were located in canonical splicing dinucleotides. The set of analyzed strains could potentially harbor additional cases of genotype-specific splicing events associated with such polymorphisms, which went undetected in our association tests due to a limited statistical power resulting from the available sample size and our conservative approach to the multiple-testing correction. Therefore we selected additional polymorphic canonical splicing dinucleotides that might have the potential to be associated with considerable changes in splicing patterns (ΔΨ > 0.1). The statistical significance of the association was calculated by Fisher’s exact test (see Methods) and then the Benjamini-Hochberg FDR < 0.01 correction was applied. In total, we found 36 genotype-specific splicing events associated with 37 sQTL at canonical dinucleotides (in addition to seven sQTL at canonical dinucleotides that had been already detected by EMMA). In one case, allele-specific inclusion of a cassette exon was associated with SNPs in canonical dinucleotides of both splice sites (a2947, Additional file 1: Table S3).

Polymorphisms at canonical dinucleotides might actually represent two different scenarios: disruption of pre-existing splice sites or creation of *de novo* splice sites. For each SNP we determined the ancestral state using orthologous genomic sequences in closely related *Drosophila* species (see Methods). In 24 of 44 cases the polymorphic splice sites represented creation of *de novo* splice sites (i.e. splice sites with ancestral not-GT/AG dinucleotides).

**Figure 3:**
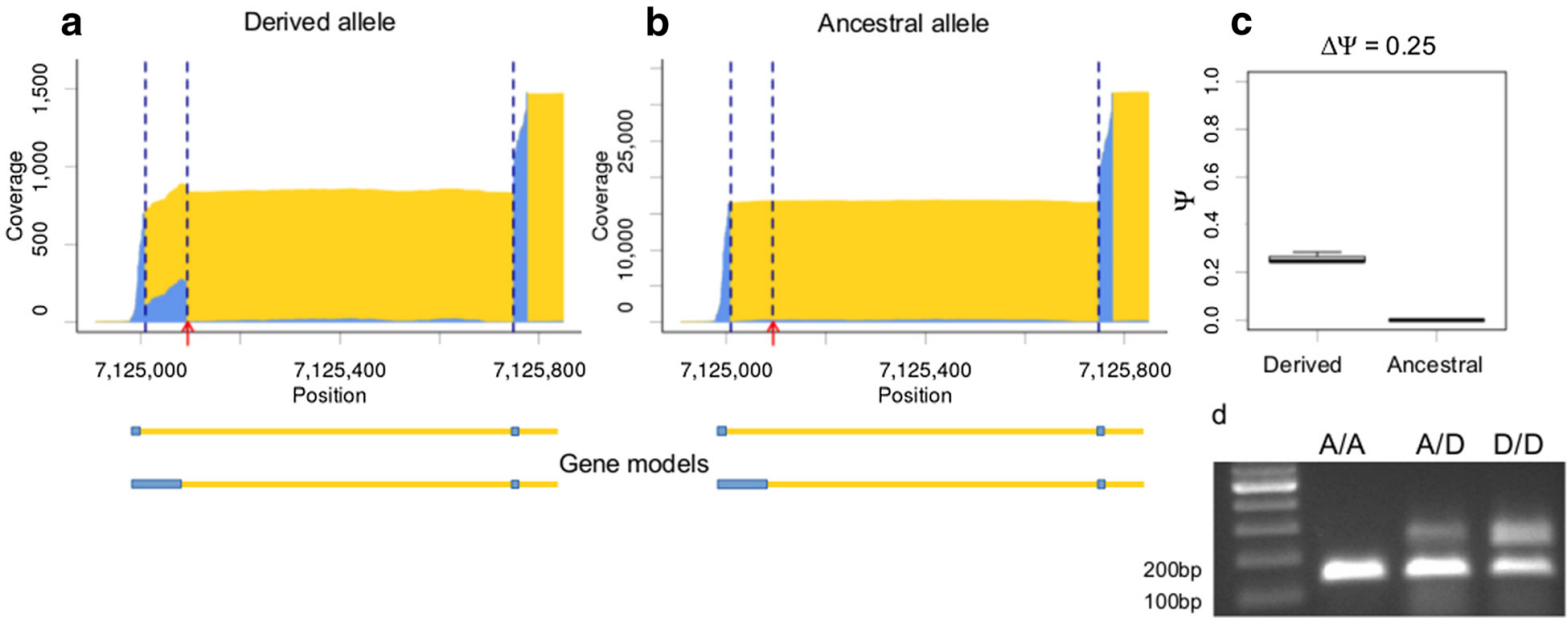
**Genotype-specific alternative donor site associated with** ***cis*** **-sQTL in canonical splicing dinucleotide.** *De novo* donor site and inclusion of an 84 bp segment that was associated with *cis*-sQTL in canonical splicing dinucleotide. **a-b:** Exon/intron coverage plots of the genotype-specific splicing event are shown for the F1-hybrids that carried derived **(a)** and ancestral **(b)** variants of *cis*-sQTL on natural alleles. Tester alleles of all F1-hybrids carried ancestral variant of *cis*-sQTL. **c:** The distributions of Ψ-values for alleles of this *cis*-sQTL. **d:** RT-PCR validation of gene models. The predicted lengths of PCR-products corresponding to the long and short isoforms were 234 bp and 150 bp, respectively. For other details see the legend to Figure 2.

An example of a genotype-specific splicing event associated with *cis*-sQTL in canonical dinucleotides is shown on Figure 3 (a1219). We observed a *de novo* donor site (mutation from GC to GT) in the first intron of the gene *dbi*. Creation of this splice site resulted in inclusion of a 84 bp intron segment to the first exon. In genotypes that carried the ancestral allele, no junction reads corresponding to this splice site were observed. The altered exon was located in the 5′-untranslated region of the gene and most probably did not affect the structure of the encoded protein. The RT-PCR validation experiment confirmed that the transcripts that utilized the new splice site (235 bp band) were expressed only in flies that carried the derived allele of *cis*-sQTL (A/D and D/D, Figure 3d). However, the homozygous D/D-inbred line was still expressing the short isoform (151 bp band), i.e. we did not observe a complete switch of isoforms.

Overall, we detected 59 genotype-specific splicing events that were associated with 104 *cis*-sQTL with ΔΨ > 0.1 (Additional file 1: Table S3).

### Allelic imbalance of *cis*-regulated genotype-specific splicing patterns

The differences in splicing patterns between the transcripts, which are expressed from two parental alleles, suggests *cis*-regulatory divergence between parental genotypes [16]. Here, we use the presence of allelic imbalance in observed genotype-specific splicing patterns as an additional evidence that the associated sQTL represent cis-regulatory variants.

Reads were aligned to parental specific reference genomes. The fraction of reads overlapping with heterozygous positions were differentially aligned to parental genome references, thus we were able to determine the allele of origin for these reads (allele-specific reads, see Methods). Allele-specific reads were used to estimate the Ψ-values separately for each parental allele, i.e. natural allele-specific (ns-Ψ) and tester allele-specific (ts-Ψ), according to the allele origin. In other words, ns-Ψ- and ts-Ψ-values represented ratios of transcripts expressed from the natural and tester alleles respectively (Figure 1a). Consequently, ns-Ψ- and ts-Ψ-values were used to measure changes in splicing patterns separately for each parental allele (ns-ΔΨ and ts-ΔΨ).

**Figure 4:**
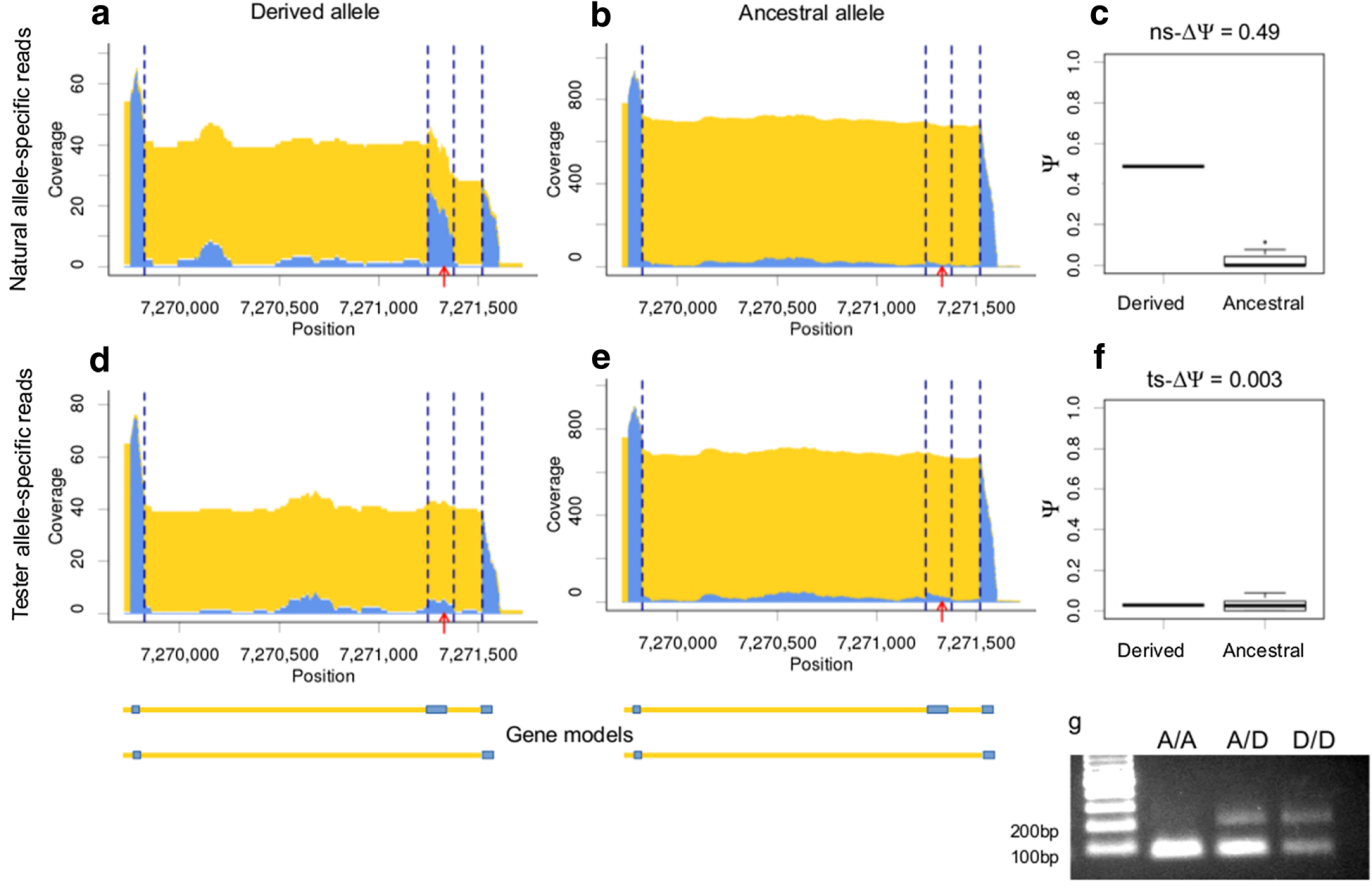
**Allelic imbalance of genotype-specific cassette exon inclusion associated with** ***cis*** **-sQTL (a625).** Genotype-specific cassette exon that exhibits strong allelic imbalance is shown. The allele-specific exon/intron coverage of genotype-specific splicing event are shown for the F1-hybrids that carried derived **(ad)** and ancestral **(be)** variants of *cis*-sQTL on natural alleles. Tester alleles of all F1-hybrids carried ancestral variant of *cis*-sQTL. The plots were created separately for the reads expressed from natural **(ab)** and tester alleles **(de)**. Only genotypes with estimated ns-Ψ and ts-Ψ-values were considered. **cf:** The distributions of ns-Ψ **(c)** and ts-Ψ-values **(c)** for alleles of *cis*-sQTL. **g:** RT-PCR validation of gene models. The predicted lengths of PCR-products corresponding to the long and short isoforms are 231 bp and 102 bp, respectively. For other details see the legend to Figure 2.

The coverage plots and distributions of Ψ-values for allele-specific reads are shown for one of genotype-specific splicing events in Figure 4. In this example, a *cis*-sQTL was associated with the inclusion of a cassette exon in the 5′-untranslated region of the gene *Sod3*. Plots were created separately for reads expressed from the natural (natural allele-specific reads, Figure 4, upper panels) and tester alleles (tester allele-specific reads, Figure 4, lower panels). Considerable inclusion of the cassette exon was observed only in transcripts expressed from natural alleles carrying the derived variant of the *cis*-sQTL (Figure 4a). At the same time, almost no inclusion of the cassette exon was detected in transcripts expressed from tester alleles carrying the ancestral variant of the *cis*-sQTL from the same F1-hybrids (Figure 4d). As a result, a considerable change in the splicing pattern associated with *cis*-sQTL is observed only for reads expressed from natural alleles (ns-ΔΨ = 0.49, Figure 4abc), and almost no change is observed for reads expressed from tester alleles (ns-ΔΨ = 0.003, Figure 4def). Strong allelic imbalance in heterozygotes implies that the observed inclusion of the cassette exon is regulated in *cis*, and that the identified *cis*-sQTL most likely is the causative regulatory variant. An RT-PCR validation experiment confirmed that observable inclusion of the cassette exon occurred only in flies that carried the derived allele of *cis*-sQTL (A/D and D/D, Figure 4g).

**Figure 5:**
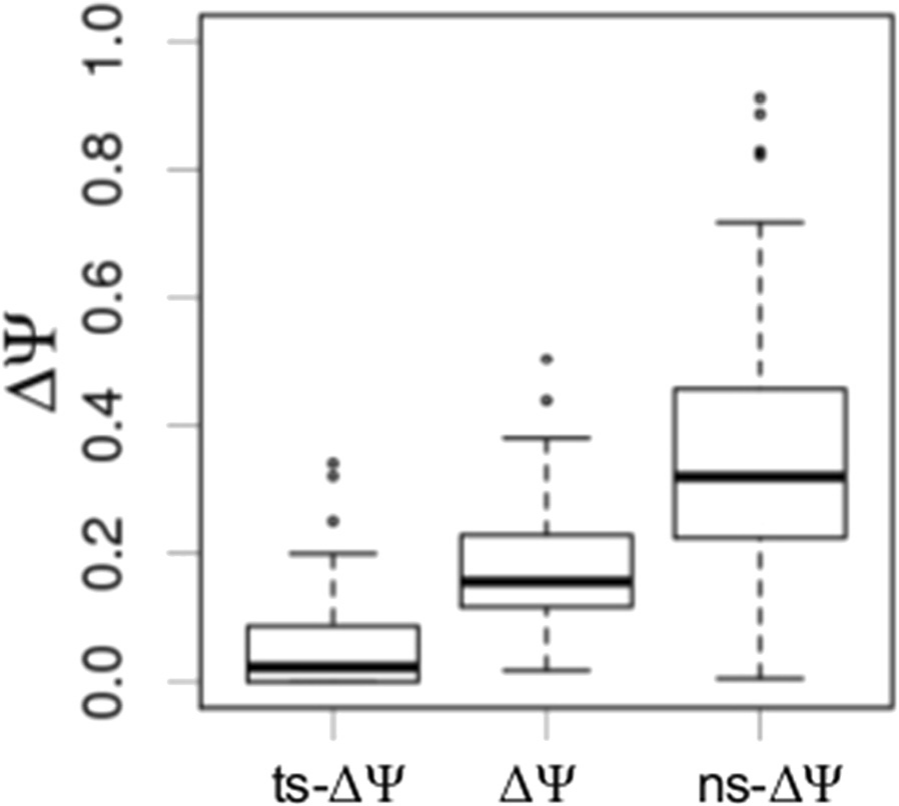
**Distributions of allele-specific ΔΨ-values.** ΔΨ-values as calculated for natural allele-specific reads **(ns-ΔΨ)**, for tester allele-specific reads **(ts-ΔΨ)** and for all reads **(ΔΨ)**. Values were calculated for *cis*-sQTL with sufficient coverage for calculation of both values for at least one F1-hybrid for each variant.

Unfortunately, due to the limited coverage by allele-specific reads, we were able to estimate ns-Ψ and ts-Ψ only for a fraction of detected genotype-specific splicing events. Both values for at least one F1-hybrid for each variant of the *cis*-sQTL was obtained for 36 *cis*-sQTL, which were associated with 17 genotype-specific splicing events. For these *cis*-sQTL, we calculated the allele-specific ΔΨ-values (ns-ΔΨ and ts-ΔΨ). The distributions of allele-specific ΔΨ-values are shown in Figure 5. On average, ns-ΔΨ-values were considerably higher than ΔΨ-values calculated for all reads, and the median of the ts-ΔΨ-values distribution is close to zero (Figure 5). In other words, almost all differences between splicing patterns associated with *cis*-sQTL are caused by changes in transcripts expressed from natural alleles. This suggests that the majority of identified genotype-specific splicing patterns indeed are regulated by *cis*-genetic effects. At the same time, in some cases allele-specific ΔΨ-values did not follow the optimal patterns that could be expected for *cis*-genetic regulation (i.e. ts-ΔΨ = 0). This might be caused by a variety of factors, including non-causative alleles, low coverage, or the influence of unknown additional genetic effects (e.g. *trans*-genetic regulation).

### Effects of genotype-specific splicing events on encoded proteins

Changes in splicing patterns may have diverse functional outcomes. For instance, they may cause exclusion or inclusion of relatively long gene segments. Moreover, such changes in protein-coding regions may result in frameshifts. Thus, mutations disrupting splice sites were often considered as loss-of-function variants [23,24].

The set of identified genotype-specific splicing events differed from the overall set of all alternative splicing event in many aspects (Table 1). Specifically, the genotype-specific changes were underrepresented among cassette exons (see Discussion). They more frequently affected terminal untranslated regions of genes (UTRs) than other alternative splicing events (66% against 29%).

**Table 1:** **Comparison of genotype-specific splicing events with the overall set of alternative splicing events**

	**Genotype-specific splicing events**	**All alternative splicing events**	**P-value (Fisher’s exact test)**
Total number of events	59	2744	
Fraction of event types			
*Alternative donor sites*	51%	46%	n/s
*Alternative acceptor sites*	42%	31%	n/s
*Cassette exons*	7%	23%	0.01
Fraction of event in UTRs	66%	29%	4.88 × 10^−9^
Fraction of frame-preserving events*	90%	78%	n/s
Median length of alternative gene segment (bp)*	9.5	20.5	2.2 × 10^−3^**
Fraction of unannotated events	75%	31%	1.45 × 10^−11^

The percents represent fractions within the “Total number of alternative splicing events”. * – In case of alternative donor/acceptor sites, the lengths of alternative gene segments were calculated as distance between alternative splice sites. This is inapplicable for terminal alternative splicing events (i.e. alternative first you set and last exons). Thus, on the estimation of the fraction of frame-preserving splicing events and median length of alternative gene segments, we exclude splicing events with more than 500 bp between alternative splice sites. ** – p-value for Wilcoxon rank-sum test.

Only 20 of 59 genotype-specific splicing events overlapped with protein-coding regions. Almost all of them were frame-preserving with lengths of alternatively spliced segments being a multiple of 3 (18 of 20). The affected gene segments were significantly shorter than that in the set of other alternative splicing events. In 14 of 20 cases genotype-specific changes involved only four or less amino acid residues (3, 6, 8 or 12 bp), including eight cases affecting only one amino acid residue (inclusion/exclusion of 3 bp).

In two cases, the lengths of altered gene segments in the protein-coding region was not a multiple of 3 and most likely lead to frameshifts. These frame-shifts disrupted protein sequences of the gene *CG31205* that encodes peptidase-S (shown in Figure 2, [25]) and the gene *nimB4* which is involved in innate immunity [26]. These cases probably represents loss-of-function variants. Interestingly, the most significant *cis*-sQTL associated with a frameshift in the gene *CG31205* is not a rare mutation: 11 of 81 studied inbred lines carry the derived allele (13.6%).

In most cases one of the alternate isoforms of a genotype-specific splicing event was not annotated (75%). This has been expected as the vast majority of them represented disruptions and creations of splice sites (i.e. new exon/intron junctions).

## Discussion

Here we studied natural genetic variation of gene models in *D. melanogaster*. We analyzed transcriptomes of 81 hybrid strains generated by crosses of inbred strains from natural populations to a single tester strain. We used RNA-Seq to annotate and to quantify alternative splicing events. Firstly, we ran association tests between isoform usage with adjacent SNPs, and identified several dozen *cis*-sQTL. Most of them were associated with mutations of regulated splice sites. Additionally, we searched for splice sites with polymorphic canonical splicing dinucleotides that were associated with considerable differences in splicing patterns. These dinucleotides are known to be crucial for the splicing mechanism and SNPs at these positions are usually considered to be regulatory variants causing the observed changes. Overall, we identified 59 genotype-specific splicing events that were associated with 104 *cis*-sQTL. Allele-specific estimates of splicing patterns confirm that the observed changes in splicing patterns are predominantly regulated in *cis*. The affected transcripts are expressed from the natural alleles rather than the tester alleles. Several representative examples were validated by RT-PCR experiments (Figures 2f, 3d, 4g).

*Cis*-sQTL located outside of the splice site region are of particular interest as they may represent mutations in auxiliary elements of the splicing code [1]. Two examples of such cases are shown in Figures 2 and 4. In the first example, four closely linked mutations are associated with the activation of a cryptic splice site located 20 bp downstream of the most significant *cis*-sQTL. In the second example, inclusion of a cassette exon is associated with a single *cis*-sQTL located in the middle of the regulated exon.

The relatively small number of genotypes and a rank-based transformation of splicing estimates resulted in a limited power of association tests. Further, the observed differences in the splicing patterns among the analyzed genotypes were probably diluted by the effects of common tester alleles. Moreover, we focused on two most basic types of alternative splicing events, thus excluding a considerable fraction of intron retentions and complex splicing events. Hence, the identified set of genotype-specific splicing events likely represents only a fraction of splicing variation existing among analyzed genotypes.

Currently, sQTL-studies are mainly focused on changes in ratios of known isoforms [13,14]. We used previously unknown high-confident splicing junctions to annotate new gene models. More than half of splice sites with SNPs at canonical splicing dinucleotides are *de novo* splice sites. Furthermore, 75% of the identified genotype-specific splicing events were not annotated, i.e. at least one of the alternate isoforms was not present in the known annotations of gene models. All these demonstrates the importance of considering qualitative changes in genotype-specific splicing patterns.

Cassette exons are underrepresented among the genotype-specific splicing events we have identified. At the same time, a comparison of alternative splicing among *D. melanogaster* lines and between closely related *Drosophila* species has revealed that cassette exons are the most divergent type of alternative splicing events [19]. The study of McManus and colleagues was focused on detection of differences in splicing patterns not considering association with sQTL. They analyzed the allelic imbalance in the interspecies F1-hybrids and showed that the regulation of cassette exons (exon skipping events) had a larger component of *trans*-genetic effects than other types of alternative splicing events [19]. On the other hand, the main focus of our study was the detection of changes in splicing patterns associated with *cis*-sQTL, and it may explain the discrepancy between our results.

The analysis of putative functional effects of identified *cis*-sQTL on gene function is consistent with results of previous studies [15,19]. In most cases, the changes in splicing patterns have no or little effect on the encoded protein sequences. On the other hand, it should be noted that the analyzed natural lines underwent many generations of inbreeding, and the majority of deleterious variants were probably washed out.

This study may be used as a prototype to design similar experiments in the future (e.g. the common reference design; allele-specific alignments; annotation of new gene models). The current bottleneck is the absence of powerful tools for such association studies, simultaneously accounting for non-normally distributed traits (e.g. isoform ratios) and population structure.

## Conclusions

The overall results of this study suggest that gene models in natural populations of *Drosophila melanogaster* may be flexible. Association tests between genotype-specific splicing patterns and SNPs identified dozens of putative splicing regulatory variants (sQTL). A set of mutations disrupting canonical dinucleotides was analyzed to identify sQTL that were not detected in the association tests. In total, we identified 59 genotype-specific splicing events that were associated with 104 *cis*-sQTL. Effects of the observed changes in gene models on the encoded proteins were predicted to be modest. The splicing polymorphisms with strong functional effects are likely selected against in natural populations, and only those with weak functional roles are segregating. The obtained results expand our understanding of how the exon/intron structure of genes evolves. The concepts applied in this manuscript may be used to design similar experiments in the future.

## Methods

### Genotypes

Genomic data for 216 *D. melanogaster* lines from two resequencing projects [20,21] were downloaded from NCBI SRA (PRJNA36679; PRJNA74721) and SNPs were called as described in ([21], Lehmann et al. manuscript submitted). We extracted SNPs that were polymorphic among 81 lines used in this study (Additional file 1: Table S1). For further analysis we used biallelic SNPs, each variant of which was homozygous in at least one line.

The ancestral states of SNPs were determined using Multiz whole-genome alignments of 14 insects from the UCSC genome browser [27,28]. An allele that was present in the orthologous position of *D. simulans* or *D. yakuba* genomes was considered as ancestral.

### Transcriptomes

The RNA-Seq data was generated from adult female heads of 81 *D. melanogaster* heterozygous crosses. F1-hybrids were obtained by crossing inbred natural lines with known genotypes with a single common tester line (*w[1118]*) (Figure 1a). Flies were cultured at 25°C on a standard dextrose/cornmeal/yeast/agar media [29]. Hybrid flies were collected onto a standard yeast/sucrose media [30]. Fly population densities were controlled by collecting eggs from population cages and pipetting equal number of eggs into culture bottles. Total RNA was extracted from adult female heads using TRIzol reagent (Life Technologies). mRNA was purified using the Dynabeads® mRNA purification kit (Invitrogen Dynal AS, Oslo, Norway). Libraries were prepared as described in [31] and sequenced on the Illumina HiSeq2000 platform (2 × 100-bp). The RNA-Seq data is available at the NCBI SRA (PRJNA74721) [(Lehmann et al. manuscript submitted)]. In the present study, multiple samples corresponding to the same F1-hybrids were merged together to achieve higher coverage per genotype.

### Allele-specific alignments

For each F1-hybrid we created two parental genome references. To this end, we updated the *Drosophila* reference genome (dm3/BDGP 5.72) with SNPs from the parental lines (tester and natural lines). We used only biallelic SNPs that were called and were in homozygous state in both parental genomes. Thus, for each F1-hybrid we created two separate parental genomes (for natural and tester lines) by update of the common reference genome at the same set of genomic positions. This allowed us to avoid spurious allelic imbalance in the case when the genomic coverage of parental genotypes differed substantially.

RNA-Seq reads were aligned to the parental genomes using STAR [32]. Alignments were run with providing known gene models (BDGP 5.72). The fraction of uniquely concordantly mapped reads varied across genotypes with an average of 71% (from 31% to 92%). Only uniquely and concordantly mapped paired-end reads were used in the analysis. We removed read duplicates using Picard MarkDuplicates (http://broadinstitute.github.io/picard/).

Most of the reads mapped to the both parental genomes with same number of mismatches. In most of the cases, two alignments of the read with the same number of mismatches to the both parental genomes are identical, but potentially they may represent mapping to different positions. Thus, we randomly choose one of them. For each read that was aligned to the two parental genome references with different number of mismatches, the alignment with fewer mismatches was selected. For reads that were aligned to only one of the parental genomes we used the obtained alignment. All the selected alignments (one alignment per read) were used to annotate and quantify splicing events.

Additionally, the reads that differentially aligned between two parental genomes (aligned to only one of the genomes or with a different number of mismatches) were considered as allele-specific reads. The reads, which aligned to the genome of a natural line with fewer mismatches, were considered to be natural allele-specific reads and were used to estimate ns-Ψ-values. The reads aligned to the genome of the tester line with fewer mismatches, were considered to be tester-allele specific reads and were used to estimate ts-Ψ-values.

The obtained fraction of allele-specific reads was less than that for interspecies hybrids [19,33] and it comprised 23% of all reads on average (from 16% to 29% per genotype). This is expected due to lower sequence divergence between alleles within a species. The allele-specific reads were used for estimation of allele-specific Ψ-values (see below).

### Annotation and quantification of alternative splicing events

Reads aligned to splicing junctions (SJs), were used to annotate and quantify alternative splicing events. We extended the set of annotated SJs (BDGP 5.72) with high-confident unannotated SJs, which shared at least one of splice sites with annotated introns. Most of them had low coverage and are likely to represent splicing errors, captured due to the high overall coverage in our data set. We filtered out weakly covered introns and kept SJs that were covered by at least 10 junction reads in at least one analyzed F1-hybrid. The majority of unannotated SJs together with parts of annotated introns were excluded by this filtering and the remaining set contained 43571 introns of protein-coding genes (Additional file 1: Table S2).

The filtered set of SJs was used to annotate two types of alternative splicing events, alternative splice sites (donor/acceptor) and cassette exons (Figure 1b). We also considered cases with multiple alternative splice sites, when the number of alternative sites was more than two. In these cases we selected two representative isoforms with the highest total coverage among all lines. For each alternative splicing event we selected representative inclusion and exclusion junctions, and we used them to quantify the isoform usage. In the case of alternative splice sites, SJs corresponding to the longer and the shorter isoforms were considered as inclusion and exclusion junctions, respectively. In the case of cassette exons, we considered one of two inclusion SJs of cassette exons, having higher coverage, as the inclusion junction. SJ corresponding to the exclusion of cassette exon was considered as the exclusion junction (Figure 1b). Reads covering inclusion (i) and exclusion (e) junctions were used to calculate Ψ-values (PSI - Percent Spliced In), which was defined as Ψ = i/(i + e). Ψ-values were calculated for each alternative splicing event in each F1-hybrid. As for both alternative splice sites and cassette exons, the inclusion junctions corresponded to the longer isoform, Ψ-value represents the inclusion level of an alternative gene segment. Ψ-values were estimated only in cases when the sum of inclusion and exclusion reads was at least 20, otherwise is considered to be not available (NA) in a given sample (F1-hybrid). Similarly, we calculated allele-specific Ψ-values (ns-Ψ and ts-Ψ) using only allele-specific reads. We annotated all alternative splicing events with identifiers from “a1” to “a2975” (Additional file 1: Table S3).

After estimation of Ψ-values we additionally filtered out alternative splicing events with almost constitutive splicing patterns in all samples (Additional file 1: Table S2). In particular, we excluded cases, with Ψ-values more than 0.9 or less than 0.1 in all analyzed genotypes.

### *cis*-sQTL mapping

Association tests between Ψ-values and SNPs were performed using EMMA implemented in R [22]. IBS kinship matrix was calculated based on SNPs called in all analyzed genotypes using the same package. SNPs had two binary encoded possible states of natural alleles (regardless to the state of SNPs in the tester allele). EMMA is an implementation of a linear mixed models and designed to handle data with normally distributed quantitative traits. Violation of this assumption often leads to inflation of false positives associations. To account for this, we applied a rank-based inverse normal transformation of Ψ-values. Ranks for Ψ-values in ties were assigned randomly. The association analysis was performed for alternative splicing events with Ψ-values estimated in at least 20 F1-hybrids. We tested *cis*-SNPs that were located in the alternative splicing event region and in 500 bp upstream or downstream.

To test for potential inflation of false-positive results we calculated genome-wide inflation factor (λ). We obtained this value for each of the detected genotype-specific splicing event, by performing association tests with 10000 random SNPs from other chromosomes. Then, λ was estimated as the ratio of medians of the observed and expected uniform log-p-value distributions.

Statistical significance of associations between variants in canonical splicing dinucleotides and changes in splicing patterns was calculated using Fisher’s exact test. To this end, we constructed 2 by 2 contingency tables for inclusion and exclusion reads for two alternative alleles of tested SNPs. Read counts from all genotypes that carried the same allele and had estimated Ψ-values were merged.

### RT-PCR validation

RT-PCR validations were performed for several representative examples of genotype-specific splicing events that affected long gene segments. For each example, we selected one heterozygous F1-hybrid that carried both derived and ancestral alleles of associated *cis*-sQTL. RT-PCR was performed for F1-hybrids and for their parental lines (i.e. the inbred tester line and the corresponding natural inbred line).

Primers were designed such that PCR products would cover alternative segments of an analyzed genotype-specific splicing events. Total RNA from adult female heads was reverse transcribed using SuperScript III First-Strand Synthesis SuperMix (Invitrogen). PCR was performed using PCR Master Mix (2X) (Thermo Scientific). PCR products were confirmed by running in 1.5% agarose gel and by UV-inspection. Images of gels were photographed by Gel Doc XR+ System (BIO-RAD). List of F1-hybrids, primer sequences and specific annealing conditions are provided in Additional file 1: Table S4.

### Availability of supporting data

Genomic and transcriptomic data analyzed in this study is available at NCBI SRA (PRJNA36679; PRJNA74721).

## Additional file

Additional file 1: Table S1. List of *D. melanogaster* strains used in the study. **Table S2.** Sets of splicing junctions (introns) and the alternative splicing events. Splicing junction were filtered by coverage (minimal coverage of 10 reads in at least one analyzed genotype). Splicing events were filtered by Ψ-values (Ψ-values less than 0.9 and more than 0.1 in at least one genotype). **Table S3.** List of identified genotype-specific splicing events and associated sQTL. * – p-values for EMMA and λ-values are available only for sQTL detected by EMMA. ** – p-values for Fisher’s exact test (FET) are available only for sQTL in canonical splicing dinucleotides. *** – total number of inclusion (*i*) and exclusion (*e*) reads across all F1-hybrids. **Table S4.** Primer sequences and specific annealing conditions for RT-PCR experiments.
